# Extracting Teeth for Pregnant Females

**Published:** 1868-05

**Authors:** 


*Query 2nd.*—"Is it consistent with sound practice to extract teeth for pregnant females?" "What is right in such cases?"

*Answer.*—Miscarriage may occur from a number of causes, and among such as may be termed *accidental* is the shock experienced from the extraction of a tooth.

Some females are very prone to this accident, such causes as mental emotions, laughing, straining, coughing, &c., bringing on the expulsive action of the uterus, while others may experience severe injuries, be far advanced in fatal diseases, and yet suffer no interruption to gestation. Although abortion, (a term used to signify a miscarriage before the sixth month, such on accident subsequent to this period being termed premature labor) is liable to occur at any period of gestation, yet it is more so at or before the third month, on account of the slight connection between the ovum and uterus. It is also very liable to occur at a time in each month corresponding to the menstrual period.

Although a miscarriage is seldom dangerous without it is accompanied with severe hemorrhage, yet the health of the female almost invariably suffers ; and this being the case, to say nothing of the unpleasant effect such an accident would produce were it to occur in a dental office, due care should be exercised by the dental practitioner in these cases. It is much better to depend upon palliative treatment than to run the risk of a miscarriage from such a violent shock as might be occasioned by the extraction of a tooth. Where the case is such as to demand the extraction of a tooth, proper inquiry should be made as to whether a proneness to this accident exists ; the family physician should be consulted and the dental practitioner be governed by his opinion.

---

